# Oral Tongue Squamous Cell Carcinoma in Young Adults in Brazil: Temporal Trends From 2013 to 2023

**DOI:** 10.1111/odi.70203

**Published:** 2026-01-18

**Authors:** Natália Santos Barcelos, Yohana Cordeiro de Miranda Magno, Juliana Maria Braga Sclauser, Renata de Castro Martins, Rodnei Alves Marques, Maria Cássia Ferreira de Aguiar, Patrícia Carlos Caldeira

**Affiliations:** ^1^ Department of Oral Pathology and Surgery, School of Dentistry Universidade Federal de Minas Gerais Belo Horizonte Brazil; ^2^ Department of Community and Preventive Dentistry, School of Dentistry Universidade Federal de Minas Gerais Belo Horizonte Brazil; ^3^ School of Technology Instituto Federal de Minas Gerais Santa Luzia Brazil

**Keywords:** Brazil, squamous cell carcinoma, tongue, young adult

## Abstract

**Background:**

This study aimed to analyze the temporal trends of oral tongue squamous cell carcinoma (OTSCC) in Brazil from 2013 to 2023, comparing young adults (20 to 44 years) to older adults (≥ 45 years).

**Methods:**

Sex, age, staging, year, and Federative Unit of diagnosis were evaluated for all cases registered under the ICD‐C02 code in a public nationwide database. Statistics encompassed the Dickey‐Fuller and Mann‐Kendall tests, Kendall's Tau coefficient, and Sen's Slope Estimator.

**Results:**

The registry of OTSCC increased from 2013 to 2023, with a stronger and more consistent trend in young adults. OTSCC showed a stronger, more consistent, and higher rate of increase in young females than in young males. OTSCC was diagnosed at advanced stages in both age groups. OTSCC increasing trends were pronounced in the North region.

**Conclusion:**

This study presented an overview of the temporal trends of OTSCC in Brazil, evidencing an increase among young women.

## 1 Introduction

Oral cavity and lip cancer represent 2% of all cancers diagnosed globally, with approximately 389,485 new cases and 188,230 deaths estimated for 2022 (Bray et al. 2024). In men, oral cavity and lip cancer is the third most incident cancer in countries with lower human development index, considering age‐standardized rates (Bray et al. 2024). Squamous cell carcinoma accounts for nearly 90% of oral cavity cancers and usually affects men over 50 years old, with smoking and alcohol consumption habits (Heller et al. 2023; Chamoli et al. 2021; Warnakulasuriya and Kerr 2021).

Recent studies have shown an increasing incidence of squamous cell carcinoma of the oral tongue (OTSCC) in young adults (≤ 45 years old), especially women without the usual risk factors of smoking and alcohol use (Annertz et al. 2012; Goldemberg et al. 2018; Jonasson et al. 2023; Jones et al. 2023; Ng et al. 2016; Satgunaseelan et al. 2020). Most of these studies encompassed countries from the northern hemisphere only, and other studies failed to find this increase in young adults (Ferreira e Costa et al. 2022; Kwon et al. 2021).

Previous studies on oral cancer in young adults in Brazil (Curioso et al. 2024; Goldemberg et al. 2018; Ribeiro et al. 2019; Souto et al. 2024) mostly pointed to a prevalence in men, with the tongue as the main affected site. However, these studies evaluated different anatomical sites altogether (tongue, base of the tongue, palate, floor of the mouth, lips, and oropharynx), some of which are known to have different etiologies. Oral cavity tumors are usually associated with tobacco and alcohol use; oropharynx/base of the tongue tumors may be associated with HPV infection, and lip tumors are related to chronic exposure to ultraviolet radiation. In addition, the data have been collected from diverse sources, usually with limited sample sizes, except for the study by Goldemberg et al. (2018).

Brazil is a country of continental dimension, with notable socio‐cultural diversity. The Unified Health System (SUS) is a national public healthcare system, freely available for all Brazilian citizens, being the single source of health care for about 80% of the population (Milani et al. 2021). From 2023 to 2025, the Brazilian National Cancer Institute estimated about 15,100 new cases of oral cavity cancer per year, being 10,900 in men and 4200 in women (Instituto Nacional de Câncer 2022).

Considering the morbidity and mortality of oral cancer; the growing evidence of an increase in OTSCC in young adults in diverse countries; and the incipient data on this regard in Brazil, the aim of this study was to analyze the temporal trends of OTSCC (ICD C02) diagnosed in young adults (aged between 20 and 44 years old) in Brazil from 2013 to 2023, compared with older adults (≥ 45 years old). The rationale is to provide a population‐based nation‐wide temporal analysis from Brazil, which can widen the understanding of OTSCC epidemiology at a global perspective.

## 2 Materials and Methods

### 2.1 Study Population

This is an analytical, population‐based, longitudinal ecological study, conducted using a public domain database. Approval from a Human Research Ethics Committee was not required as data under analysis are open access, secondary, and did not allow individualization.

The registries of oral tongue squamous cell carcinoma (OTSCC) diagnosed in the Brazilian population were obtained from the Oncology Treatment Monitoring Panel (Panel‐Oncology: http://tabnet.datasus.gov.br/cgi/dhdat.exe?PAINEL_ONCO/PAINEL_ONCOLOGIABR.def). This is an open public database with nationwide coverage, provided by the Informatics Department of the Unified Health System (DATASUS). The database compiles data on diagnostic and therapeutic procedures carried out by public and private healthcare institutions that offer cancer treatment in Brazil and are accredited by the national Unified Health System (SUS).

The inclusion criteria comprised all records available at Panel‐Oncology under the ICD‐C02 code (malignant neoplasm of other and unspecified parts of tongue). Cases of patients aged < 20 years were excluded. Information was collected based on the Federative Unit where the OTSCC diagnosis was made, considering all 27 Brazilian Federative Units. The records were stratified by: year of diagnosis (from 2013 to 2023), sex (female, male), age range (20 to 44 years or ≥ 45 years), and staging (TNM 0, I, II, III, IV). All records were collected in duplicate by two researchers (N.S.B. and Y.C.M.M) on October 25, 2024.

To evaluate the number of OTSCC registries per 100,000 inhabitants in each Brazilian geographic region from 2013 to 2023, we used the 2000–2070 population projections from the Brazilian Institute of Geography and Statistics (IBGE), available at https://www.ibge.gov.br/estatisticas/sociais/populacao/9109‐projecao‐da‐populacao.html (accessed on March 25, 2025).

### 2.2 Statistical Analysis

The gathered data were organized in a Microsoft Excel database. Statistical analysis was performed using R software, version 4.4.0 (R Core Team 2024; R Foundation for Statistical Computing, Vienna, Austria). The Dickey‐Fuller test was used to assess the stationarity of time series; the Mann‐Kendall test was applied to detect monotonic upward or downward trends, with Kendall's Tau coefficient used to determine the strength and consistency of the trend; and Sen's Slope Estimator was used to estimate the magnitude and direction of trends over time. *p*‐values < 0.05 were considered statistically significant.

## 3 Results

**FIGURE 1:**
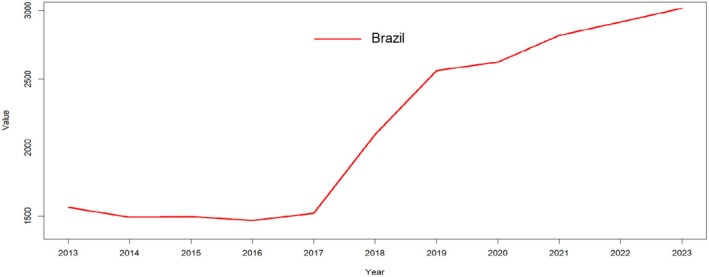
Number of OTSCC cases registered in individuals ≥ 20 years‐old in Brazil from 2013 to 2023.

From 2013 to 2023, a total of 23,583 cases of OTSCC were registered in adults over 20 years old in Brazil. In the nationwide time series analysis, considering all individuals over 20 years of age, the Dickey‐Fuller test indicated non‐stationarity (*p* = 0.3549), while the Mann‐Kendall test revealed a statistically significant upward monotonic trend (*p* = 0.0010). The Sen's Slope Estimator was 177.8 (95% CI = 106.4–241.0) and the Tau was 0.782. From 2017 onwards, a marked increase in the number of OTSCC registries was observed (Figure 1).

### 3.1 The Registry of OTSCC Increased in Brazil From 2013 to 2023, With a Stronger and More Consistent Trend in Young Adults

**FIGURE 2:**
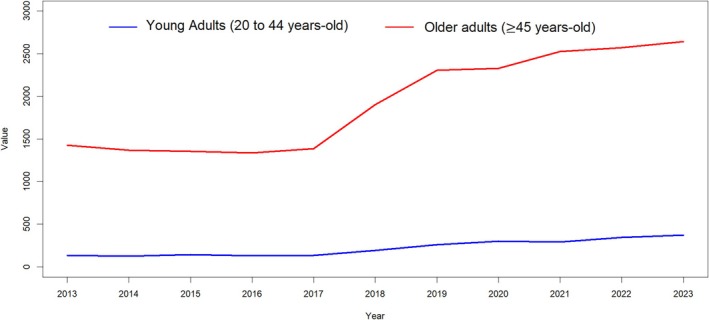
Number of OTSCC cases registered in Brazil from 2013 to 2023, comparing young adults (20 to 44 years old) and older adults (≥ 45 years old).

The number of registries of OTSCC showed a non‐stationary, upward monotonic temporal trend from 2013 to 2023 for both age groups, as indicated by the Dickey‐Fuller and Mann‐Kendall tests, respectively (*p* = 0.1073 and *p* = 0.0010 for young adults; *p* = 0.4261 and *p* = 0.0018 for older adults). The growth rate was higher in individuals aged ≥ 45 years (Sen's Slope Estimator = 148.2, 95% CI = 84.7–205.3) compared to young adults (Sen's Slope Estimator = 27.5, 95% CI = 18.0–36.2). Nonetheless the trend was stronger and more consistent in young adults (Tau = 0.782) than in older adults (Tau = 0.745) (Figure 2).

### 3.2 In Young Adults, OTSCC Shows a Stronger, More Consistent, and Higher Rate of Increase in Females Than in Males

**FIGURE 3:**
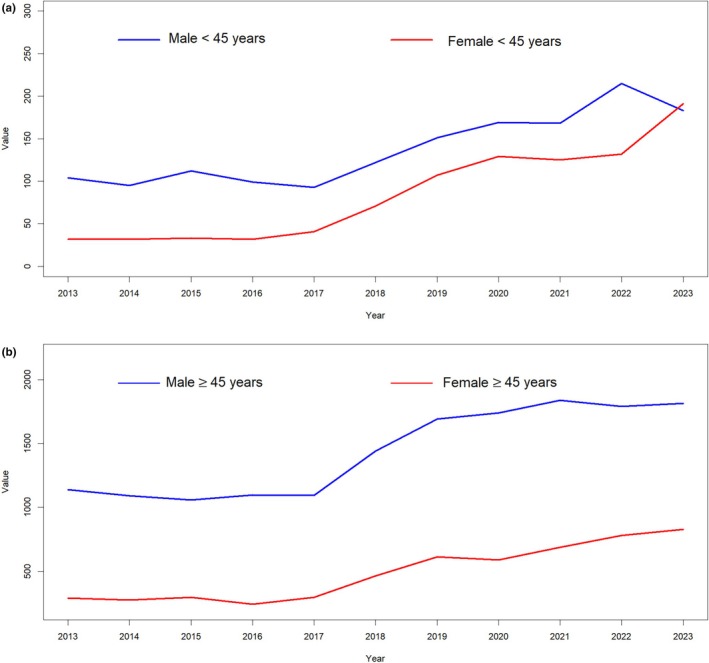
Number of OTSCC cases registered in Brazil from 2013 to 2023, stratified by sex. Image (a) refers to young adults (20 to 44 years old). Image (b) refers to older adults (≥ 45 years old).

For young adults, the time series of both female and male were non‐stationary (Dickey‐Fuller test: *p* = 0.5548 males, *p* = 0.6139 females), exhibiting significant upward monotonic trends (Mann‐Kendall test: *p* = 0.0050 males, *p* = 0.0002 females), with a marked increase after 2017. The increase was stronger, more consistent, and with a higher rate in female individuals (Sen's Slope Estimator = 15.3, 95% CI = 9.0–21.0; Tau = 0.898) than in males (Sen's Slope Estimator = 11.2, 95% CI = 6.7‐17.3; Tau = 0.673), with a marked convergence of the curves from 2023 on (Figure 3a).

In older adults, the time series were non‐stationary among males and stationary among females (Dickey‐Fuller test: *p* = 0.5224 males; *p* = 0.0289 females). However, both sexes showed a significant monotonic upward trend (Mann‐Kendall test: *p* = 0.0030 males; *p* = 0.0010 females). The number of OTSCC registries was higher among males throughout the study period (Sen's Slope Estimator = 87.5; 95% CI = 30.7–130.1; Tau = 0.709); nevertheless, the growth rate was stronger and more consistent among females (Sen's Slope Estimator = 63; 95% CI = 49.7–86.7; Tau = 0.782) (Figure 3b).

Finally, the trend was stronger and more consistent among young females (Tau = 0.898) compared to older females (Tau = 0.782). However, among males, the trend was stronger and more consistent in those aged ≥ 45 years (Tau = 0.709) than in younger adults (Tau = 0.673).

### 3.3 OTSCC Is Diagnosed at Advanced Stages in Both, Young and Older Adults

Across all age groups, TNM stages 0, I, and II showed lower incidence, while most records were staged III and IV (Figure S1).

Among young adults, all TNM stage series, except TNM III, were non‐stationary (Dickey‐Fuller test: 0 = 0.8974; I = 0.7967; II = 0.3040; III = 0.0100; IV = 0.2668), and none showed a monotonic trend (Mann‐Kendall test: 0 = 0.6920; I = 0.1010; II = 0.2628; III = 0.0724; IV = 0.8755). The Tau coefficients were 0 = 0.114, I = −0.404, II = −0.289, III = 0.44, IV = 0.055.

For older adults, all TNM stage series were non‐stationary (Dickey‐Fuller test: 0 = 0.1224; I = 0.8721; II = 0.2546; III = 0.7499; IV = 0.3639). With the exception of stages I and IV, they exhibited monotonic trends (Mann‐Kendall test: 0 = 0.0417; I = 0.0509; II = 0.0002; III = 0.0010; IV = 0.8147). Stages 0 and II showed significant negative trends (Tau = −0.5 and −0.881, respectively), while stage III showed a significant positive trend, with growth during this period (Tau = 0.782).

### 3.4 Among the Five Geographic Regions of Brazil, OTSCC Trends Were Most Pronounced in the North

Considering the frequency of OTSCC registries per 100,000 inhabitants in each Brazilian geographic region, regardless of age and sex, all time series were non‐stationary (Dickey‐Fuller test: North = 0.9087; Northeast = 0.1495; Southeast = 0.4806; Midwest = 0.1353; South = 0.469), and an inflection point in the upward trend of registries was observed since 2017/2018. The North region presented the highest growth rate (Sen's Slope Estimator = 0.2042, 95% CI = 0.0518–0.3451), with a consistent and strong positive trend (Tau = 0.745) (Figure S2).

When analyzing OTSCC registries in young adults by geographic region, the North region showed the highest growth rates for both sexes, with a more pronounced increase among females, whose growth was approximately 2.3 times higher than males (Sen's Slope Estimator: males = 0.076; females = 0.175). In the North, the trend was also stronger and more consistent among young females (Tau = 0.898) compared to males (Tau = 0.673). Finally, in the North region the time series were non‐stationary and monotonic for both males and females (Dickey‐Fuller test: males = 0.9868; females =0.990; Mann‐Kendall test: males = 0.005; females = 0.0002) (Figure S2).

Still regarding young adults, at the Southeast and South regions the growth rates were higher and statistically significant among females compared to males (Sen's Slope Estimator—Southeast: males = 0.010, females = 0.021; South: males = 0.011, females = 0.033). Moreover, the growth trend was stronger and more consistent in females than in males at these geographical regions, as indicated by higher Tau coefficients (Southeast: males = 0.455, females = 0.673; South: males = 0.382, females = 0.818) (Supporting Information S1).

Regarding OTSCC registries among older adults, time series were non‐stationary for all geographic regions (Dickey‐Fuller test: *p* > 0.05), indicating the presence of trends over time. The North region showed the highest significant growth rate for both sexes (Sen's Slope Estimator: male = 0.376; female = 0.533), in addition to exhibiting the strongest and most consistent upward trend among females, as indicated by the Tau coefficient (0.709) (Figure S2).

Still concerning older adults, in the Southeast and South regions, growth rates were statistically significant (*p* < 0.05) only among females, despite the higher growth rates observed in males (Sen's Slope Estimator—Southeast: male = 0.208, *p* = 0.061; female = 0.122, *p* = 0.008; South: male = 0.265, *p* = 0.212; female = 0.183, *p* = 0.012). In the Midwest, growth rates were statistically significant for both sexes (*p* < 0.05), although the rate was higher among males (Sen's Slope Estimator—male = 0.247, *p* = 0.008; female = 0.081, *p* = 0.008). Furthermore, the growth trend was stronger and more consistent among females in both Southeast (Tau: male = 0.455; female = 0.636) and South regions (Tau: male = 0.309; female = 0.600), while the Midwest region demonstrated a strong and consistent growth pattern similar for both sexes (Tau = 0.636) (Supporting Information S2).

## 4 Discussion

This study showed an increase in the registries of OTSCC in young adult females, with emphasis to the growth rates in the North region of the country. The searched database contains the official information on OTSCC diagnoses issued in the Brazilian public health system (SUS), which is managed by the Ministry of Health. Of notice, in 2018 the Oncology Treatment Monitoring Panel began to require mandatory notification for diagnoses under ICD‐10, including C02 (*Portaria SAS N° 643/2018*) (Ministério da Saúde (Brasil), Secretaria de Atenção à Saúde 2018; Ministério da Saúde, Departamento de Informática do SUS (DATASUS) 2025). In addition, SUS represents the single means of accessing healthcare services for approximately 80% of the Brazilian population (Milani et al. 2021). Therefore, we can assume the results reported herein reflect the national scenario of OTSCC in adults in Brazil over 10 years. To our knowledge, this is the first study to report such trends in Brazil.

In recent years, the increasing incidence of OTSCC in young adults has been reported in many countries (Burus et al. 2024; Ng et al. 2016; Satgunaseelan et al. 2020), such as the United States of America, Canada, Finland, Iceland, Ireland, Sweden (Ng et al. 2016), Australia (Ng et al. 2016; Satgunaseelan et al. 2020), Singapore (Ng et al. 2016; Satgunaseelan et al. 2020), France (Deneuve et al. 2021), Brazil (Goldemberg et al. 2018), and Nordic countries (Annertz et al. 2012), with a notable rise among women (Goldemberg et al. 2018; Ng et al. 2016; Satgunaseelan et al. 2020). The present study is consistent with those previous reports; however, comparisons should take into consideration some methodological variations such as the definition of age groups, tumor sites, and sample sizes. For instance, institutional‐based data may not reflect population‐based statistics.

The study of Ng et al. (2016) provided a comprehensive and broad analysis of OTSCC in countries in the northern hemisphere. After evaluating 89,212 cases of tongue squamous cell carcinoma from 22 databases in 14 countries (Australia, Austria, Bulgaria, Canada, Denmark, England, Estonia, Finland, Iceland, Ireland, Norway, Singapore, Sweden, and USA), the authors reported a higher incidence rate in young patients (< 45 years) in 14/22 registries in 8 countries (Australia, Canada, Finland, Iceland, Ireland, Singapore, Sweden, and USA). They also observed statistically significant annual increase rates among women, except in Iceland, Singapore, and some regions of the USA.

Burus et al. (2024) evaluated 58,661 oral tongue cancer records from the USCS Public Use Database (98% coverage of the USA) between 2001 and 2019. Although recognizing the increase in oral tongue cancer incidence among young adults in the USA in past decades, they indicate the increase in early‐onset cases (< 50 years) among non‐hispanic white women is shifting to a peak among individuals aged 50–59 years. Furthermore, they predict a tendency toward increased diagnoses in older individuals and a smaller discrepancy between males and females through 2034, based on birth cohort effects analysis.

Ferreira e Costa et al. (2022) evaluated the frequency of oral squamous cell carcinoma in young adults (≤ 40 years old, including children) among 10,727 biopsies from oral diagnosis centers in Brazil, India, South Africa, the United Kingdom, Argentina, Mexico, Spain, and China between 1998 and 2018. Eight oral sites including tongue were evaluated and 5.8% of the cases affected young adults. Although those authors did not show an increase in oral squamous cell carcinoma among young adults, they recognized a subgroup of young female patients with unknown exposure to risk factors as a distinct group. On this regard, Kwon et al. (2021). did not find a significant annual increase in oral cavity squamous cell carcinoma among young (< 45 years) never smoker females in South Korea from 2006 to 2016. Methodological differences concerning the data sources and sampling may explain the divergence between those studies and the present ones.

In Brazil, Souto et al. (2024) evaluated 108 patients treated for tongue squamous cell carcinoma between 2000 and 2012. The cut‐off used to classify individuals as young adults was 50 years old. Among the different sub‐sites of the tongue, such as the base of the tongue, unspecified sites, and overlapping areas, the lateral border of the tongue was the most affected site in both individuals aged 22–40 and 41–50 years. Goldemberg et al. (2018) analyzed 28,029 tongue cancer cases in Brazil between 2000 and 2012 and found an increase in the incidence of cancer at the base of the tongue, a slight increase in oral tongue cancer among women under 50 years of age, and a decrease in the incidence of this lesion in men. Although the base of the tongue was the most commonly affected site overall, among individuals under 40 years old, oral tongue cancer occurred more. Our study encompassed 23,583 cases and showed that 10 years after Goldemberg's study (Goldemberg et al. 2018), OTSCC growth rates in young adult females increased significantly and even equaled those of males by 2023.

Sartori et al. (2023) analyzed the temporal trend of mortality due to oral, oropharyngeal, and lip cancers in Brazil between 1980 and 2018. A total of 81,918 deaths were analyzed, and 18,385 were women. When stratified by geographic region, the North region showed an upward trend in the mortality rate from 1991 to 2018. There was also an increase in mortality from all cancers investigated among women between 1980 and 2018. Although they did not stratify the cases by age and included tumors from different locations and etiologies, their results seem aligned with the notable increase in the number of notifications in the North region and the rise in OTSCC cases in female young adults reported in the present study.

The stage at diagnosis influences prognosis, survival rates, treatment options, and financial issues (Milani et al. 2021), which may render devastating impacts in young adults. In the present study, individuals were mostly diagnosed at stages III and IV, regardless of age group. Accordingly, in northeastern Brazil between 1988 and 2013, among young adults (≤ 50 years‐old) with squamous cell carcinoma on the lips, oral cavity and/or oropharynx, 26/38 were staged III and IV, while 12/38 were at early stages (Ribeiro et al. 2019). However, female records showed a higher proportion of tumors in stages I and II compared to male records. Souto et al. (2024) observed similar proportions of early and advanced stages among patients aged ≤ 40 years, despite the limited sample size. Oliver et al. (2019) found that among patients < 40 years of age with OTSCC in the USA from 2004 to 2015, 1225 were diagnosed at stages I–II, while 515 were at advanced stages. Taken together, these results indicate variable conclusions about the staging at diagnosis of oral squamous cell carcinoma in young patients, and the different samples analyzed and access to healthcare may influence this variable.

The North region was the one with the highest growth rates for females and males (both, young and non‐young) in the present study. The Brazilian National Oral Health Policy (PNSB, Política Nacional de Saúde Bucal) was implemented in 2004 and increased access to dental services in vulnerable areas, as the North region (Galante et al. 2021). According to Silva et al. (2024), between 2018 and 2023, the North showed high rates of public oral health service utilization. These efforts may partially account for a higher coverage of the health‐based registries in the North region. However, in our study, we cannot determine whether this reflects a true increase in access to diagnostic services, an actual increase in the number of cases, or merely an increase in the number of records reflecting a better coverage of registries, since the period of rising cases coincides with the period of creation and mandatory reporting in the Oncology Panel. However, considering the magnitude of the increase found herein and the complexity and uniqueness of the North region of Brazil, we believe these results merit further investigations looking for possible explanations.

The reason for the increased incidence of OTSCC among young adults, including young women, remains unknown and some authors consider it a distinct clinical entity. Early exposure to tobacco and alcohol cannot be ruled out (Xu et al. 2019); however, most cases lack association with these traditional risk factors (Chatzopoulos et al. 2021; Harris et al. 2009). Chronic trauma and oral microbiome imbalance have been suggested (Tran et al. 2023), while HPV does not appear to be associated with OTSCC (Deneuve et al. 2022). The OTSCC immunohistochemical profile of apoptosis, cell cycle, cell proliferation, and angiogenesis does not seem to differ by age, and no definitive genetic etiology for oral cavity cancer in young people has been established yet (Benevenuto et al. 2012; Barnabé et al. 2019; Choi et al. 2019; Kim et al. 2023; Tran et al. 2023). Therefore, further studies investigating the etiology of OTSCC in young adults are clearly warranted. The current study could not access data on alcohol/tobacco use, habits, or comorbidities, limiting inferences on possible causality.

Despite the strengths of our data, this study has some limitations inherent to the study design and the type of records used. As an ecological study, we cannot interpret the results at the individual level, including information on risk factors and educational level. The use of secondary data may present inaccuracies due to underreporting, outdated information, or delays in records, either because of technical problems or the absence of trained professionals to perform the task (Montagnoli et al. 2024). The huge territorial dimension of Brazil and its cultural, socioeconomic, and healthcare access differences may also represent a limitation to interpretation and generalization of the results. The standardization of regional data per 100,000 inhabitants was an effort in minimizing this limitation. Finally, the rates were not adjusted for age within each group, and there is no standardized age range to classify young adults.

In conclusion, we report an upward temporal trend of OTSCC registries in young adult females, as by analyzing official national‐wide data from the Brazilian Ministry of Health. A notable increasing trend was noticed in the North region of the country, though we cannot determine whether this reflects a better coverage of records or a real increase in the number of cases. These results present the overview of the temporal trend of OTSCC in Brazil, which seems accordant with previous similar studies in other countries. Health‐promoting organizations and professionals should be aware of this changing epidemiological scenario to plan actions properly. For example, oral mucosa screening can be included in the Brazilian public policy of health care for women (Ministério da Saúde 2011), as well as for men (Ministério da Saúde 2008) [National Policy for Comprehensive Women's Health Care (PNAISM, Política Nacional de Atenção Integral à Saúde da Mulher); National Policy for Comprehensive Men's Health Care (PNAISH, Política Nacional de Atenção Integral à Saúde do Homem)]. Finally, further studies are encouraged to identify the risk factors and pathways involved in OTSCC development in young adults, especially women.

## Author Contributions

**Natália Santos Barcelos:** methodology, investigation, writing – original draft, formal analysis, data curation, conceptualization. **Yohana Cordeiro de Miranda Magno:** methodology, investigation, data curation. **Juliana Maria Braga Sclauser:** data curation, investigation, methodology. **Renata de Castro Martins:** conceptualization, investigation, methodology, validation, writing – review and editing, formal analysis. **Rodnei Alves Marques:** writing – review and editing, validation, formal analysis. **Maria Cássia Ferreira de Aguiar:** writing – review and editing, investigation. **Patrícia Carlos Caldeira:** conceptualization, investigation, writing – review and editing, validation, project administration, formal analysis, supervision, resources.

## Funding

Natália Santos Barcelos was granted a fellowship provided by ‘Coordenação de Aperfeiçoamento de Pessoal de Nível Superior’ (CAPES)/Brazil (Financial Code 001). Yohana Cordeiro de Miranda Magno was granted a fellowship provided by ‘Pró‐Reitoria de Pesquisa da Universidade Federal de Minas Gerais/Conselho Nacional de Desenvolvimento Científico e Tecnológico’ (PRPq‐UFMG/CNPq) (Grant #05/2024).

## Conflicts of Interest

The authors declare no conflicts of interest.

## Supporting information


**TABLE S1:** Temporal analysis of cases of oral tongue squamous cell carcinoma (ICD‐02) in Brazil, from 2013 to 2023. Data from adults between 20 and 44 years‐old, stratified by sex and by the Brazilian geographic regions.

**TABLE S2:** Temporal analysis of cases of oral tongue squamous cell carcinoma (ICD‐02) in Brazil, from 2013 to 2023. Data from adults over 45 years‐old, stratified by sex and by the Brazilian geographic regions.

**Figure S1:** Clinical stage (TNM) of OTSCC cases registered in Brazil from 2013 to 2023. Image (a) refers to young adults (20 to 44 years old). Image (b) refers to older adults (≥ 45 years old).

**Figure S2:** Number of OTSCC cases per 100,000 inhabitants in each geographical region of Brazil, registered from 2013 to 2023. Image (a) refers to all cases registered. Image (b) refers to females aged 20 to 44 years. Image (c) refers to males aged 20 to 44 years. Image (d) refers to females aged ≥ 45 years. Image (e) refers to males aged ≥ 45 years.

## Data Availability

The data that support the findings of this study are available in Panel‐Oncology at http://tabnet.datasus.gov.br/cgi/dhdat.exe?PAINEL_ONCO/PAINEL_ONCOLOGIABR.def, reference number ICD‐02. These data were derived from the following resources available in the public domain: Panel‐Oncology, http://tabnet.datasus.gov.br/cgi/dhdat.exe?PAINEL_ONCO/PAINEL_ONCOLOGIABR.def.
