# Supplementary material for: Oral Tongue Squamous Cell Carcinoma in Young Adults in Brazil: Temporal Trends From 2013 to 2023
Source: Oral Dis. 2026 Jan 18;32(5):1300–7. doi: 10.1111/odi.70203 (PMC13365011; doi:10.1111/odi.70203)
Supplement: Supplementary file 2 — TABLE S2: Temporal analysis of cases of oral tongue squamous cell carcinoma (ICD‐02) in Brazil, from 2013 to 2023. Data from adults over 45 years‐old, stratified by sex and by the Brazilian geographic regions. [file ODI-32-1300-s002.docx]

| **Geographic Region** | **Males** | | | **Females** | | |
| --- | --- | --- | --- | --- | --- | --- |
|  | **Dickey-Fuller** | **Mann-Kendall** | **Sen’s Slope Estimator** | **Dickey-Fuller** | **Mann-Kendall** | **Sen’s Slope Estimator** |
| North | 0.7443 | 0.0127  T = 0.6 | 0.0127  S = 0.376 | 0.8282 | 0.00309  T = 0.709 | 0.00309  S = 0.533 |
| Northeast | 0.367 | 0.2757  T = 0.273 | 0.2758  S = 0.070 | 0.3383 | 0.1194  T = 0.382 | 0.1195  S = 0.062 |
| Southest | 0.3853 | 0.0617  0.0455 | 0.0617  S = 0.0208 | 0.1831 | 0.00812  T = 0.636 | 0.00812  S = 0.122 |
| Midwest | 0.6611 | 0.008  T = 0.636 | 0.0081  S = 0.247 | 0.5301 | 0.00812  T = 0.636 | 0.0081  S = 0.081 |
| South | 0.4405 | 0.2129  T = 0.309 | 0.2129  S = 0.265 | 0.5164 | 0.0127  T = 0.6 | 0.0127  S = 0.183 |

**Supplementary material (S2)**

**Table S2. Temporal analysis of cases of oral tongue squamous cell carcinoma (ICD-02) in Brazil, from 2013 to 2023. Data from adults over 45 years-old, stratified by sex and by the Brazilian geographic regions.**
