# Supplementary figures and images for: Oral Tongue Squamous Cell Carcinoma in Young Adults in Brazil: Temporal Trends From 2013 to 2023
Source: Oral Dis. 2026 Jan 18;32(5):1300–7. doi: 10.1111/odi.70203 (PMC13365011; doi:10.1111/odi.70203)

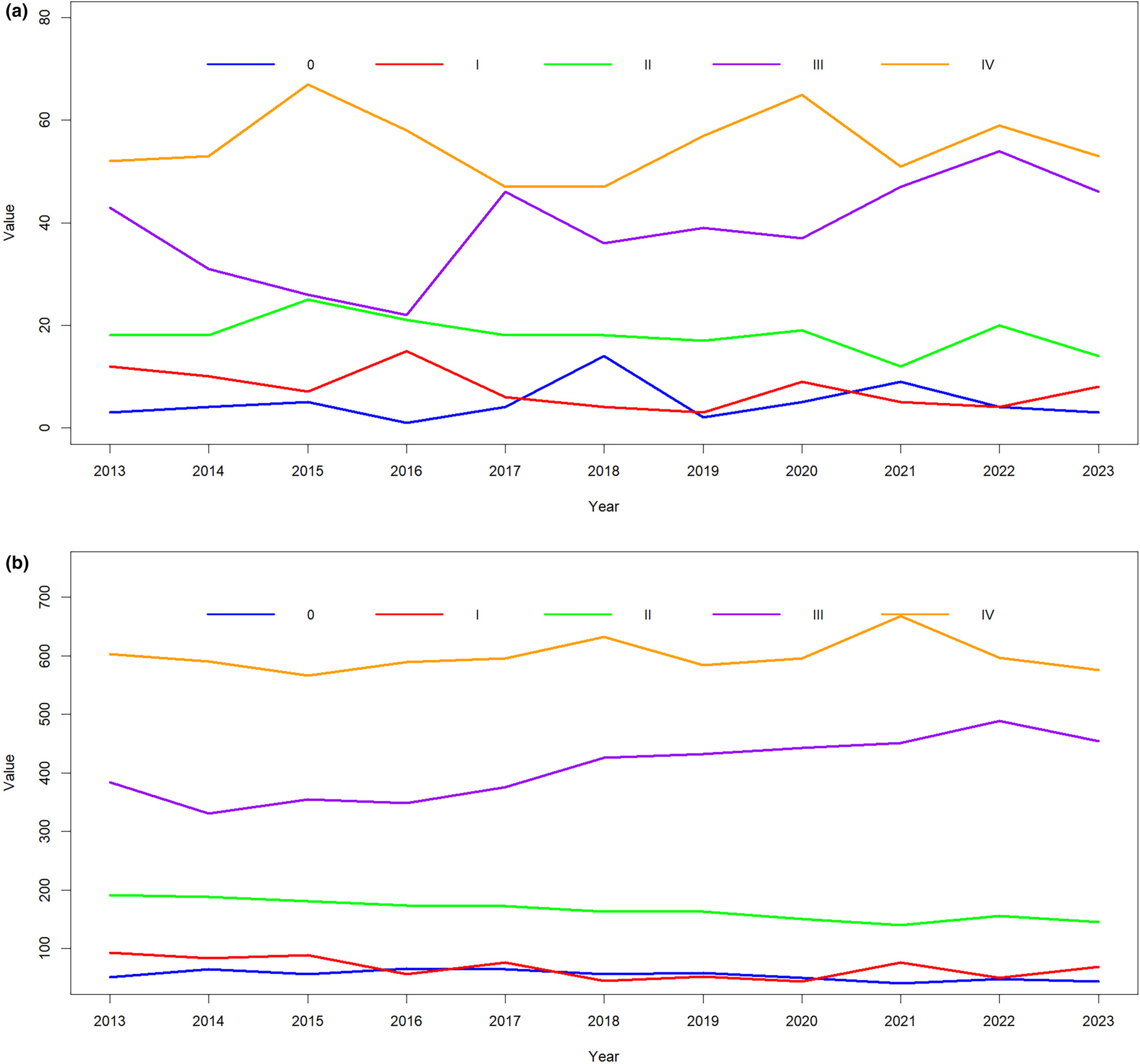

Supplement: Supplementary file 3 — Figure S1: Clinical stage (TNM) of OTSCC cases registered in Brazil from 2013 to 2023. Image (a) refers to young adults (20 to 44 years old). Image (b) refers to older adults (≥ 45 years old). [file ODI-32-1300-s003.png]

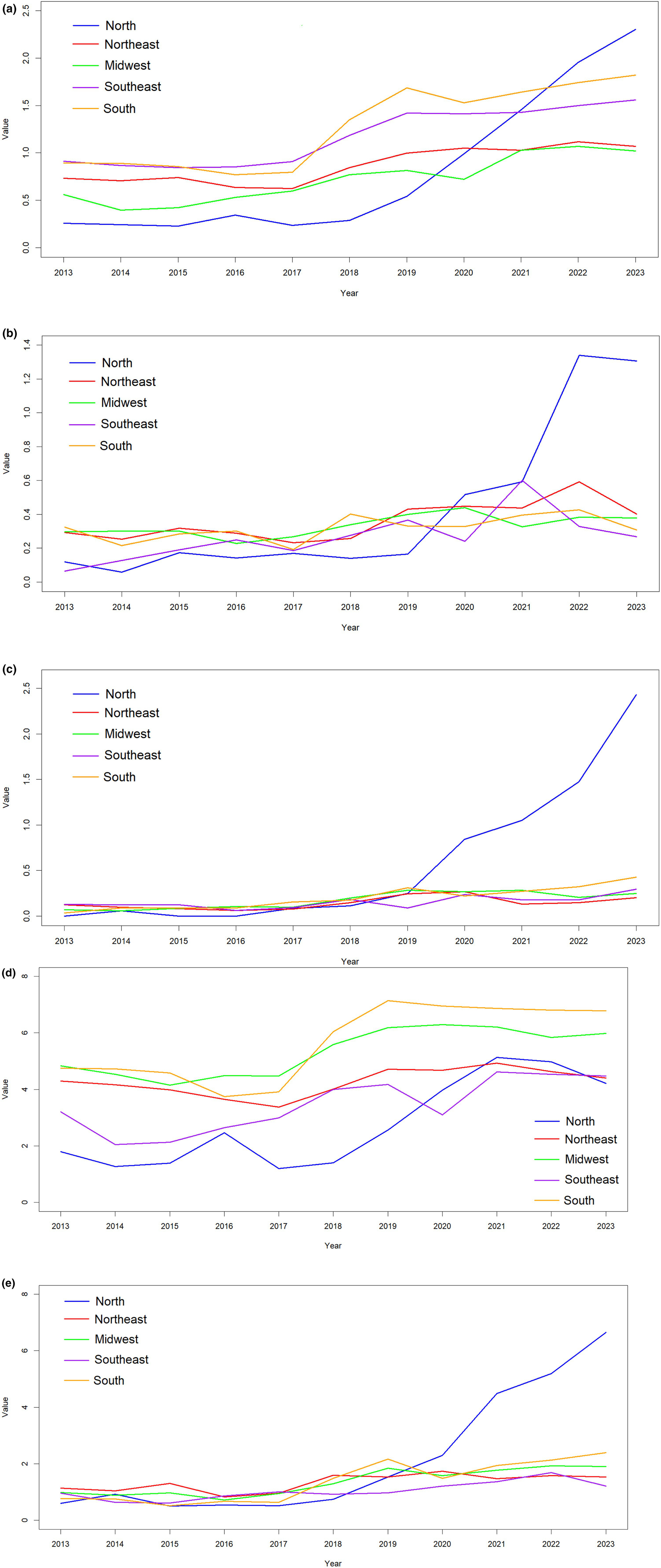

Supplement: Supplementary file 4 — Figure S2: Number of OTSCC cases per 100,000 inhabitants in each geographical region of Brazil, registered from 2013 to 2023. Image (a) refers to all cases registered. Image (b) refers to females aged 20 to 44 years. Image (c) refers to males aged 20 to 44 years. Image (d) refers to females aged ≥ 45 years. Image (e) refers to males aged ≥ 45 years. [file ODI-32-1300-s004.png]
